# Colonic Malakoplakia With an Adenomatous Appearance on Magnification Endoscopy: A Case Report

**DOI:** 10.1002/deo2.70296

**Published:** 2026-02-18

**Authors:** Shima Sono, Kiichiro Kaji, Miyabi Miura, Hideo Takayama, Kohei Yasuda, Kuniaki Arai, Kenichi Harada, Shuichi Terasaki

**Affiliations:** ^1^ Department of Gastroenterology Japanese Red Cross Kanazawa Hospital Kanazawa Ishikawa Japan; ^2^ Department of Human Pathology Graduate School of Medical Sciences Kanazawa University Kanazawa Ishikawa Japan

**Keywords:** colonic polyps, colorectal tumor, magnifying endoscopy, malakoplakia, Michaelis–Gutmann bodies

## Abstract

Malakoplakia is a rare chronic granulomatous disease associated with impaired macrophage phagocytosis of bacteria. Because colonic malakoplakia presents with varied endoscopic appearances, its characteristic features have not been clearly defined, and differentiation from colorectal neoplasms during endoscopy may be difficult. We report a case of colonic malakoplakia with adenomatous features evaluated using magnifying narrow‐band imaging (NBI). A patient receiving hemodialysis and corticosteroid therapy for immunoglobulin A nephropathy underwent total colonoscopy after a positive fecal immunochemical test. Two reddish polyps were identified in the ascending colon. Magnifying NBI showed either absent or regular surface patterns with fine, thread‐like vessels, along with areas resembling adenomatous changes. Although malignant features were not observed, a definitive diagnosis could not be established endoscopically. Consequently, both lesions were resected using cold snare polypectomy. Histopathological examination revealed a granulation tissue‐like inflammatory lesion composed of histiocytes containing Michaelis–Gutmann bodies in the lamina propria and submucosa, leading to a diagnosis of malakoplakia. Based on this diagnosis, a surveillance colonoscopy was scheduled for 1 year later.

## 1 Introduction

Malakoplakia is an uncommon chronic granulomatous inflammatory disorder characterized by defective bactericidal activity of macrophages, resulting in incomplete phagocytosis and accumulation of bacterial remnants. Although it can affect various organs, the colon is among the most frequently involved extravesical sites [1]. Because colonic malakoplakia presents with diverse endoscopic appearances, its characteristic features have not been clearly defined, and differentiation from colorectal neoplasia may be difficult [2]. In addition, the diagnostic value of magnifying endoscopy for colonic malakoplakia has not been established.

Here, we report a case of colonic malakoplakia evaluated using magnifying narrow‐band imaging (NBI).

## 2 Case Report

The patient was a man in his 70s who underwent a total colonoscopy at our hospital following a positive result on a fecal immunochemical test (FIT). His medical history included a distal gastrectomy for gastric cancer at the age of 65. He had been diagnosed with IgA nephropathy 39 years earlier and had been on maintenance hemodialysis for 14 years. Twelve years before presentation, he developed adrenal insufficiency and dumping syndrome, for which he had been receiving oral corticosteroid therapy for 11 years.

He reported a smoking history of 20 cigarettes per day, discontinued around the age of 40, and moderate alcohol consumption. Three years before the current presentation, total colonoscopy performed after a positive FIT had revealed colonic adenomas, which were endoscopically resected.

**FIGURE 1:**
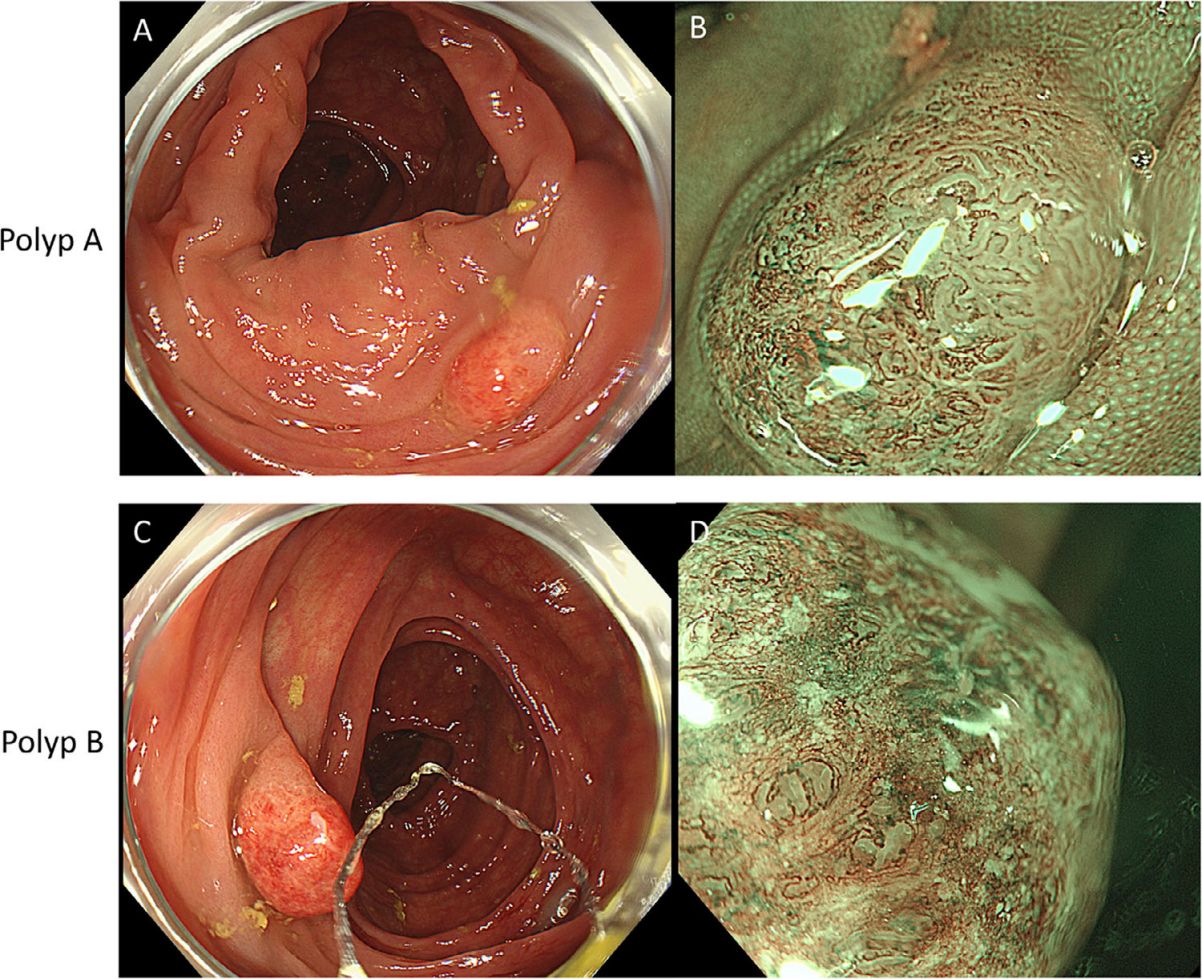
Two reddish polyps were identified during colonoscopy. (A, C) Both polyps, A and B, appeared erythematous upon gross inspection. (B) Upon narrow‐band imaging (NBI) magnification of polyp A, an adenomatous architecture was observed in approximately half of the lesion, whereas the remaining portion demonstrated loss of glandular structures accompanied by a crimped vascular network. (D) In polyp B, the glandular architecture was almost absent, and a similarly crimped vascular pattern was evident.

In all, nine polyps were detected during colonoscopy. Using NBI magnifying endoscopy and the NBI magnifying endoscopic classification of colorectal tumors proposed by the Japan NBI expert team (JNET) criteria [3], seven polyps were diagnosed as adenomas. Endoscopic diagnosis of the remaining two reddish polyps (designated polyps A and B) in the ascending colon was challenging (Figure 1). Polyp A was estimated to have a maximum diameter of 5 mm. Approximately half of its surface exhibited adenomatous features and was classified as JNET type 2A. The remaining half demonstrated thin, thread‐like, tortuous microvessels, with the surface either lacking a discernible structure or showing a regular pattern. Polyp B was estimated to have a maximum diameter of 10 mm and was largely devoid of a discernible surface structure. Its microvasculature was thin and tortuous, resembling the non‐adenomatous portion of polyp A. Although no malignant features were observed, endoscopic findings such as mucosal hyperemia and loss of surface structure in polyps A and B raised the possibility of inflammatory polyps, either alone or in combination with adenomas. Based on these findings, all polyps were removed using cold snare polypectomy.

**FIGURE 2:**
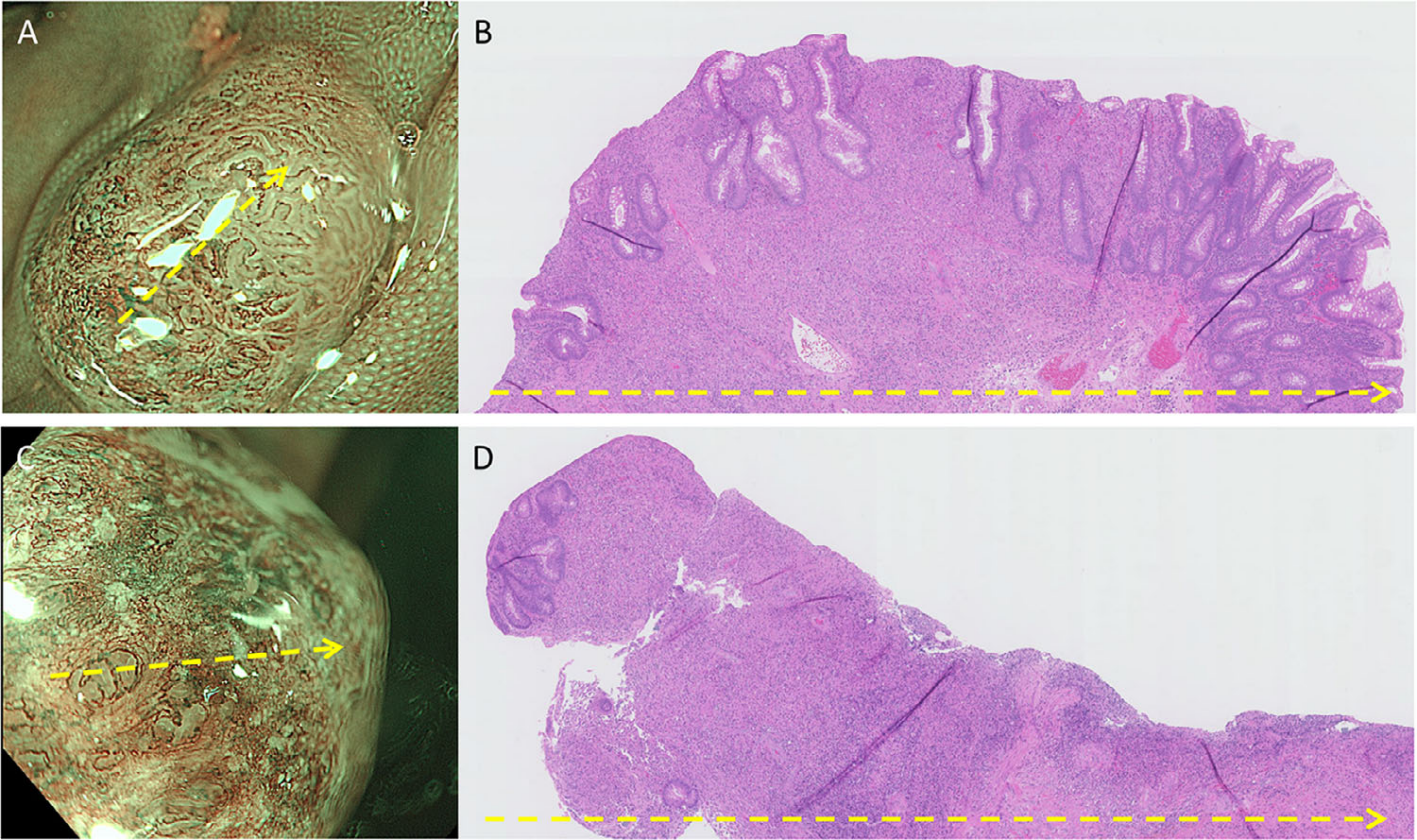
Histopathological findings versus narrow‐band imaging (NBI) magnifying imaging results of polyps A and B. NBI magnifying imaging of (A) polyp A and (C) polyp B. (B) The cut surface of polyp A, sectioned along the direction indicated by the arrow in (A), exhibits glandular structures with mild atypia in the area endoscopically identified as adenomatous. In contrast, the non‐adenomatous region contained only sparse glandular structures. (D) The cut surface of polyp B revealed few glandular structures and features suggestive of mucosal surface desquamation, similar to the NBI magnifying imaging.

**FIGURE 3:**
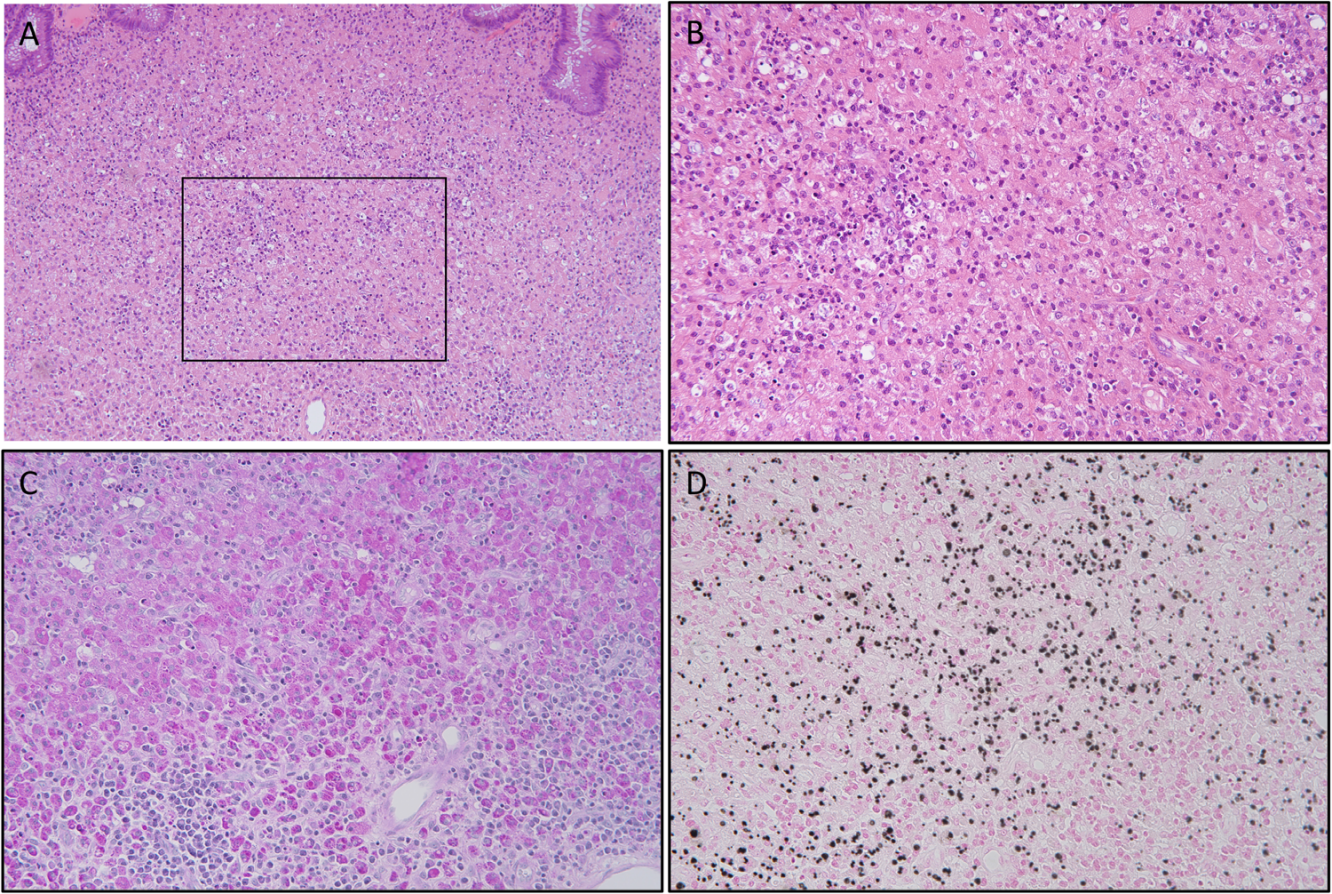
Lamina propria of polyp A. (A) Hematoxylin and eosin staining demonstrates granulation tissue‐like inflammatory lesions. (B) Magnified view of the area outlined by the black box in (A). (C, D) PAS and von Kossa staining of the same region depicted in (B), highlighting Michaelis–Gutmann bodies, which are positive in both stains.

Figure 2 shows the correlation between histopathological findings and NBI magnifying imaging in polyps A and B. The cut surface of polyp A, sectioned along the direction indicated by the arrow, revealed glandular structures with mild atypia in the area endoscopically identified as adenomatous, while the non‐adenomatous regions exhibited few glandular structures in the mucosal surface. Histologically, the adenoma‐like portion of polyp A was a tubular adenoma with low‐grade dysplasia. The cut surface of polyp B showed almost no glandular architecture; instead, it exhibited dense cellular infiltration and features suggestive of mucosal surface desquamation. In both polyps, inflammatory foci resembling granulation tissue and containing numerous histiocytes were observed extending from the lamina propria into the submucosa (Figure 3). Additionally, layered concentric structures (Michaelis–Gutmann [MG] bodies) that were positive for PAS and von Kossa staining were identified. Based on these histopathological findings, both polyps A and B were diagnosed as malakoplakia.

Because the patient had no gastrointestinal symptoms, bacterial infection was not suspected. Accordingly, no microbiological tests were performed, and no additional examinations or treatments were undertaken. A surveillance colonoscopy was scheduled for 1 year later.

## 3 Discussion

Colonic malakoplakia is the most common gastrointestinal form, accounting for just over 60% of the cases (14 of 23) reported by Lee et al. [2] and 88% of the cases (23 of 26) reported by Zhang et al. [4]. Both studies noted that colonic malakoplakia most frequently affects the sigmoid colon and rectum. In contrast, involvement of the ascending colon, as observed in the present case, is rare. The predilection for specific colonic segments is thought to be related to differences in the intestinal microbiota between the ascending colon and the distal colon (sigmoid colon and rectum). Malakoplakia has been associated with Gram‐negative bacilli, such as *Escherichia coli* [2, 4], and Enterobacteriaceae are reportedly abundant in the rectal mucosa of affected patients [5]. These microbiota differences may influence the predilection sites of malakoplakia, although further investigation is required.

Colonic malakoplakia can present as polyps, masses, or erythema. The differential diagnosis is broad, including neoplastic lesions, inflammatory bowel disease, intestinal tuberculosis, granulomatous diseases, histiocytic and giant cell lesions (e.g., sarcoidosis), Whipple's disease, and Langerhans cell histiocytosis [2]. Malakoplakia lesions predominantly occur in the lamina propria rather than the mucosal surface and may present as subepithelial lesions [6, 7]. These features contribute to the difficulty of endoscopic diagnosis.

Because NBI magnification is primarily used to examine the mucosal surface, its utility in diagnosing colonic malakoplakia has been presumed to be limited. However, in the present case, it revealed both glandular structures indicative of adenomatous components and areas suggestive of inflammatory changes in polyp A, while most glandular structures were absent in polyp B. Previous reports of colonic malakoplakia presenting as subepithelial lesions have described lesions smaller than in the present case, typically measuring 3–5 mm in diameter. By contrast, polyp B in our patient was larger than polyp A. Although polyp A may represent a “collision lesion”, in which malakoplakia incidentally occurs at the base of an adenoma [8], it is difficult to interpret the minimal residual glandular structures observed in polyp B as a collision lesion. The differing appearances of polyps A and B, both representing colonic malakoplakia, may be explained by differences in the degree of inflammatory cell infiltration and surface architectural exfoliation related to lesion size. However, this hypothesis cannot be fully substantiated based on the present report alone, and further investigation is required. Nevertheless, the current report provides valuable insight into the findings of NBI magnification in colonic malakoplakia.

One of the important roles of magnifying endoscopy in the evaluation of colorectal polypoid lesions is that of differentiating neoplastic lesions from inflammatory lesions. An unstructured surface has been reported as an endoscopic feature of early, poorly differentiated colorectal adenocarcinoma [9], and distinction from malakoplakia may therefore be challenging in some cases. For small lesions such as those in the present case, biopsy may have a substantial impact on the lesion because of biopsy‐induced scarring, and there are additional concerns regarding patient burden associated with repeat total colonoscopy if further endoscopic resection becomes necessary. In light of these considerations, we believe that, in this case, endoscopic mucosal resection rather than cold snare polypectomy should have been selected to achieve reliable and complete resection. Overall, careful magnifying endoscopic evaluation with NBI is required when differentiating colorectal neoplasms from inflammatory lesions, including colonic malakoplakia, because poorly differentiated colorectal adenocarcinoma may be difficult to distinguish from inflammatory changes.

Colonic malakoplakia requires surgical intervention if the disease extends beyond the colon, if significant symptoms are present, or if malignancy cannot be excluded [10]. When confined to the colon, the prognosis is generally favorable, and the condition can often be managed effectively with endoscopic resection and antibiotic therapy [2]. In the present case, given the absence of symptoms and the complete endoscopic resection of malakoplakia confined to the large intestine, additional antibiotic therapy was deemed unnecessary. There is no consensus regarding the optimal interval for endoscopic follow‐up after treatment. Additional case accumulation will be necessary to establish evidence‐based guidelines for follow‐up scheduling.

In conclusion, although endoscopic diagnosis of colonic malakoplakia remains challenging, NBI magnifying endoscopy can provide valuable information for identifying features that differ from typical adenomas (JNET type 2A), thereby helping to avoid missed lesions and to guide decisions regarding biopsy or resection.

## Author Contributions

**Conceptualization**: Shima Sono, Kiichiro Kaji, and Miyabi Miura. **Supervision**: Kuniaki Arai and Kenichi Harada. **Visualization**: Kiichiro Kaji, Shima Sono, Miyabi Miura, and Kenichi Harada. **Writing – original draft**: Kiichiro Kaji. **Writing – review & editing**: All authors.

## Conflicts of Interest

The authors declare no conflicts of interest.

## Funding

None.

## Ethics Statement

All procedures were conducted in accordance with the principles of the 1964 Declaration of Helsinki and its subsequent amendments.

## Consent

Informed consent was obtained from the patient and his family.
